# On genetic diversity in caraway: Genotyping of a large germplasm collection

**DOI:** 10.1371/journal.pone.0244666

**Published:** 2020-12-29

**Authors:** Daniel von Maydell, Heike Lehnert, Thomas Berner, Evelyn Klocke, Wolfram Junghanns, Jens Keilwagen, Frank Marthe

**Affiliations:** 1 Institute for Breeding Research on Horticultural Crops, Institute of Federal Research Centre for Cultivated Plants, Julius Kuehn-Institute, Quedlinburg, Germany; 2 Institute for Biosafety in Plant Biotechnology, Institute of Federal Research Centre for Cultivated Plants, Julius Kuehn-Institute, Quedlinburg, Germany; 3 Dr. Junghanns GmbH, Aschersleben, Groß-Schierstedt, Germany; Chinese Academy of Sciences, CHINA

## Abstract

Caraway (*Carum carvi*) is a widespread and frequently used spice and medicinal plant with a long history of cultivation. However, due to ongoing climatic changes, the cultivation is becoming increasingly risky. To secure caraway cultivation in future, timely breeding efforts to develop adapted material are necessary. Analysis of genetic diversity can accompany this process, for instance, by revealing untapped gene pools. Here, we analyzed 137 accessions using genotyping by sequencing (GBS). Hence, we can report a broad overview of population structure and genetic diversity of caraway. Population structure was determined using a principal coordinate analysis, a Bayesian clustering analysis, phylogenetic trees and a neighbor network based on 13,155 SNPs. Genotypic data indicate a clear separation of accessions into two subpopulations, which correlates with the flowering type (annual *vs*. biennial). Four winter-annual accessions were closer related to biennial accessions. In an analysis of molecular variance, genetic variation between the two subpopulations was 7.84%. In addition, we estimated the genome size for 35 accessions by flow cytometry. An average genome size of 4.282 pg/2C (± 0.0096 S.E.) was estimated. Therefore, we suggest a significantly smaller genome size than stated in literature.

## Introduction

Caraway (*Carum carvi* L., 2n = 2x = 20 [1]) is a herb of the Apiaceae family (syn. Umbelliferae). It is widely distributed in Europe, Asia and North Africa and was introduced to America and New Zealand [2]. Caraway fruits are used as spice and as pharmaceutical to ease gastrointestinal afflictions [3]. There is evidence for usage in ancient times [3]. Besides the health benefits, caraway cultivation positively affects crop rotation [4] and provides nourishment for a variety of insects [5,6]. Therefore, caraway cultivation can be a valuable component of agrobiodiversity.

Since the medieval period, biennial ecotypes of caraway have been cultivated from Italy to Scandinavia [3]. Until today, biennial varieties are in cultivation. Annual varieties were only introduced in Europe in the 1990s [3]. Due to on-going climatic changes, the cultivation becomes riskier. For instance, in 2018 and 2019, drought stress events negatively affected caraway cultivation in Germany. The evaluation of phenotypic diversity of available caraway germplasm in combination with pre-breeding efforts will help to provide new caraway genotypes which are well adapted to changing growing conditions. This will contribute to a less risky cultivation of caraway in the future. The analysis of population structure and genetic diversity can accompany this process, for instance, by revealing untapped gene pools.

Until now, only a few studies dealt with the molecular genetics of caraway. These studies used randomly amplified polymorphic DNA (RAPD) markers and treated small sets from 5 to 24 accessions [7–9]. Based on 56 polymorphic bands (RAPD), Laribi *et al*. [7] conducted distance-based methods (unweighted pair group method of arithmetic means (UPGMA) and a factorial correspondence analysis (FCA)) to reveal the population structure of five annual caraway accessions. Seidler-Lozykowska *et al*. [9] investigated the population structure of 24 biennial accessions using UPGMA, based on 23 polymorphic bands (RAPD). As for most minor crops, no reference genome is available for caraway. RAPD markers can be used for diverse species without prior knowledge on sequences [10]. Disadvantages are that mostly dominant markers are detected and that results are difficult to reproduce [10,11].

The primary goal of this study was to detect population structure and genetic diversity within a large set of caraway accessions. This set should represent a majority of available caraway germplasm and not a small selection of genotypes as in prior studies. In addition, we asked whether population structure could be correlated with available data on the accessions like flowering type, classification, geographic origin, national breeding programs or germplasm provider. Furthermore, we wanted to know whether domestication and breeding reduced the genetic diversity in caraway cultivars. For this purpose, we wanted to implement a genome-wide and cost-effective method, which requires no reference genome and allows simultaneous genotyping of a vast number of genotypes. Due to protandry, caraway is expected to be predominantly an outcrossing species [1,12]. Hence, a high proportion of heterozygosity was expected for several caraway accessions. Therefore, ideally the chosen genotyping method should also allow the detection of heterozygous loci.

Genotyping by sequencing (GBS) fulfills all these requirements. This next generation sequencing (NGS) method has already been implemented for a variety of crops, including horticultural species, with the aim of investigating population structure and genetic diversity [13–16]. GBS usually leads to the discovery of a vast number of single nucleotide polymorphism (SNP) loci [17,18]. Because these SNPs could also be used for further applications like genome-wide association studies (GWAS) or the development of diagnostic markers, GBS can also be a very sustainable method for caraway breeding research.

Knowledge of genome size is advantageous before conducting a GBS. In particular, genome size plays a role for the choice of the restriction enzyme. Das [19] published a genome size of 4C = 19.051 pg for one caraway accession by Feulgen cytophotometry. Feulgen cytophotometry (or densitometry) is based on indirectly measuring stain absorbance [20]. Flow cytometry, as an alternative method, is based on measuring stain fluorescence [20]. Flow cytometry is an efficient and accurate method for genome size estimation [20–23], which was already used for some species of the Apiaceae family [24–26]. Investigations ahead of this study indicated that Das [19] clearly overestimated the genome size of caraway. Therefore, as an additional study, we aimed to re-estimate the genome size of caraway by flow cytometry.

## Material and methods

### Germplasm

A set of 137 caraway accessions was assembled from gene banks, breeding companies and institutes: The Leibniz Institute of Plant Genetics and Crop Plant Research (IPK, Gatersleben, Germany; 30 accessions) and the United States Department of Agriculture (USDA, Ames, USA; 17 accessions) each provided all their available caraway accessions. Accessions from the Crop Research Institute (CRI, Prague, Czech Republic; 14 accessions), the Centre for Genetic Resources, the Netherlands (CGN, Wageningen, Netherlands; 6 accessions) and the Nordic Genetic Resource Center (NordGen, Alnarp, Sweden; 4 accessions) were added to cover additional countries or cultivars. Further breeding material and cultivars were provided by the Julius Kuehn-Institute (JKI, Quedlinburg, Germany; 48 accessions), by Dr. Junghanns GmbH (Ascherleben, Germany; 7 accessions), by Agritec Ltd. (Šumperk, Czech Republic; 6 accessions), by the National Research Council Canada (NRC, Ottawa, Canada; 2 accessions), by Chrestensen GmbH (Erfurt, Germany; 2 accessions) and by Pharmasaat GmbH (Artern, Germany; 1 accession). In total, the accessions originated from 27 countries (collecting or breeding site). **Table 1** provides a summary assigning accessions to classification and flowering type. Detailed information on accessions is provided in **S1 Table**.

**Table 1:** Summary of 137 caraway accessions used in this study.

	Flowering type		
Classification	Biennial	Annual	Total
Cultivars	19	8	27
Breeding material	1	54	55
Landrace	12	2	14
Wild	29	2	31
Unknown	9	1	10
**Total**	**70**	**67**	**137**

Accessions were assigned to classification groups according to the information supplied by the germplasm provider. The flowering type was verified in a field trial at the JKI in 2018 and/or 2019.

Due to the assumed outcrossing nature of caraway, we expected most biennial varieties and wild material to be highly heterozygous populations. This should also be true for annual varieties. In contrast, annual breeding material of the JKI and Dr. Junghanns GmbH was produced by inbreeding. It mainly originated from crossings between annual and biennial material and was selected for annual flowering. The breeding lines are in the fourth or fifth generation of inbreeding (n = 27 and n = 24, respectively). Two annual breeding lines are classified as doubled haploid (DH) lines. These lines were produced by the NRC using microspore culture [27]. For two accessions, classified as breeding material (one biennial and one annual accession from Agritec Ltd.), more detailed classification is not available.

### Flowering type

In May 2018, pre-grown plants (early leaf stage with up to six developed leaves, BBCH 13–16 [28]) were planted in field in a randomized complete block design. For each accession, up to three repetitions were planted in small plots each containing 25 plants in a distance of 10 cm. Single plants were considered as flowering with the opening of the first umbel (BBCH 61 [28]). Accessions were considered as annual flowering types if the majority of individual plants per plot flowered in the first year of cultivation. All other accessions were considered as biennial flowering types. For seven accessions, which could not be planted in 2018, the flowering type was evaluated in 2019. No complete verification of the flowering type was carried out for annual breeding material of the JKI, which was already investigated in previous years and was known to consist of annual flowering types only. The number of repetitions per accession and year is provided in **S1 Table**.

### Genome size

A set of 35 accessions was selected for genome size estimation. This set includes accessions out of all classification groups and flowering types (**S1 Table)**. Three individual plants per accession were grown in greenhouse and were sampled at medium leaf-stage with six or more developed leaves (BBCH 16–19, Hack *et al*. [28]). The samples were analyzed using the flow cytometer FACSCalibur (BD Biosciences). The tomato (*Solanum lycopersicum*) cultivar 'Stupické' was used as reference standard of known genome size (2C = 1.96 pg) [29]. Leaf fragments of the sample and the reference standard were simultaneously cut with a razor blade. The CyStain PI Absolute P reagent kit (Sysmex) was used for extraction and staining. The factor of 0.978 Gb = 1 pg was used for conversion [30]. Tests of significance were computed using a paired t-test in R software [31].

### Genotyping by sequencing

Plant material for DNA extraction was grown under greenhouse conditions in two replicates for each accession. Young leaf material (BBCH 13–16 [28]) of five individual plants per replicate was sampled and pooled, so that each accession ideally is represented by ten individual plants in two pools.–This was done to maintain the complex genetic information stored in highly heterozygous populations.–DNA was extracted using DNeasy Plant Mini Kit (Qiagen). DNA quality and quantity were tested using the Qubit 2.0 Fluorometer (Life Technologies) and gel electrophoresis.

GBS was carried out by Elshire Group Ltd. (Palmerston North, New Zealand). For GBS analysis, the method published by Elshire *et al*. [17] was adapted to caraway: 100 ng of genomic DNA and 1.44 ng of total adapters were used; the genomic DNAs were restricted with EcoT22i enzyme; the libraries were amplified with 18 PCR cycles. The replicates were split up into two libraries, the first containing 185 and the second 89 replicates. The libraries were sequenced using HiSeq X Ten (Illumina) generating 150 bp paired end reads.

### SNP discovery

The platform Galaxy [32] was employed to process raw sequences and to perform clustering and SNP calling. GBS raw reads were trimmed using Trim Galore (v0.4.0; non-default parameters: quality 30, length 50). Afterwards the reads of the two replicates per accession were merged. Because a reference genome of caraway is not available, a reference was built using vsearch (v2.7.1_linux_x86_64) including a dereplication (default parameter) and a clustering (non-default parameter: cluster_fast, id 0.93, sizein True, sizeout True) [33]. In detail, the built reference can be called a 'mock reference', composed of consensus GBS fragments [34]. Reads were mapped against this reference using BWA-mem (v0.7.15-r1140) [35]. SNP calling was carried out using samtools mpileup (v1.2 using htslib 1.2.1; non-default parameters: output-tags DP,DPR) [36]. SNPs were filtered for quality, ≤ 10% missing values, ≤ 5% minor allele frequency (MAF) and ≤ 90% maximum heterozygosity.

In total, the number of reads per genotype after trimming ranged between 1,722,352 and 14,361,058 with an average number of 5,621,037 reads. The clustering resulted in 1,196,473 contigs with an average length of 145.56 bp. The SNP calling generated 13,155 SNPs after filtering. On average, this set contained 4.42% missing values per accession and at maximum 16.19%. Detailed values per accession are provided in **S1 Table**.

### Population structure and genetic diversity

Bayesian clustering analysis was performed using STRUCTURE (v2.3.4) [37–40]. A putative number of K = 1–10 subpopulations was tested within ten independent runs. Markov Chain Monte Carlo (MCMC) iterations and the number of burn-ins were set to 50,000. The output was analyzed using Structure harvester (v0.6.94) [41]. To select the optimal number of K, the Evanno ΔK method was applied [42]. Caraway accessions were assigned to a subpopulation according to a membership coefficient of > 0.5. PCoA was conducted using Darwin (v6.0.14) [43]. For visualization of population structure, the R package ggplot2 was used [31,44]. Phylogenetic trees were built using the R packages adegenet [45,46] and ape [47]. In detail, the algorithm BIONJ [48] was used for the final NJ tree. Ten thousand bootstraps were conducted and nodes with < 30% support were dissolved to multifurcations. To reveal possible phylogenetic net structures between accessions, a neighbor network was created using the software SplitsTree (v4.15.1) [49].

Allele frequencies and observed (H_O_) and expected heterozygosity (H_E_) were calculated using R software [31]. Tests of significance were computed using a paired t-test in R software [31]. An analysis of molecular variance (AMOVA) was conducted using the software Arlequin (v3.5.2.2) [50]. By default, loci with > 5% missing values were excluded.

## Results

### Flowering types

In total, 67 accessions were found to be annual flowering types, whereas 70 accessions were found to be biennial flowering types. In several plots of the biennial accessions, a few individual plants started flowering in the first year, but late, between the end of August and October. These accessions showed only small inflorescences and no seeds were produced.

### Genome size

**Fig 1:**
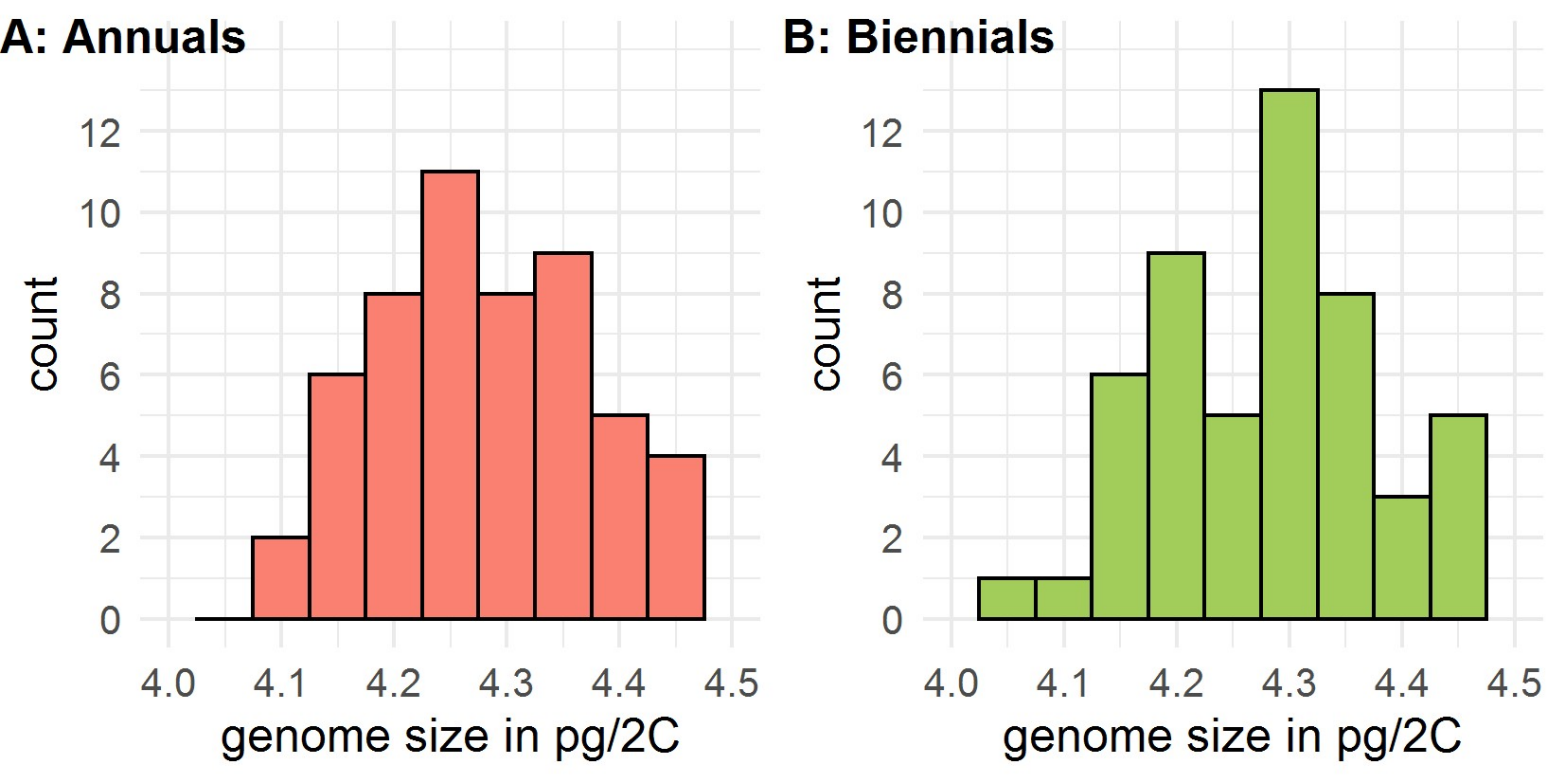
Estimated genome size (in pg/2C) of caraway based on flow cytometric analysis of 35 accessions. Three biological replicates per accession were investigated.

Across all measured samples, an average genome size of 4.282 pg/2C (± 0.0096 S.E.) was estimated within a range from 4.051 to 4.482 pg/2C (**Fig 1**). Annual flowering types (n = 54) had a genome size of 4.285 pg/2C (± 0.013 S.E.) and biennial flowering types (n = 51) of 4.279 pg/2C (± 0.014 S.E.). This small difference was not significant (t = 0.30446, df = 102.36, p-value = 0.7614). An estimated mean value for the genome size of each measured accession is given in **S1 Table**.

### Population structure and genetic diversity

The population structure of the 137 caraway accessions was determined using a PCoA, a Bayesian clustering analysis, phylogenetic trees and a neighbor network based on 13,155 SNPs. Further analysis included AMOVA and the estimation of H_O_ and H_E_, to analyze genetic diversity of the defined subpopulations and within some subsets of interest. For orientation, we defined five subgroups, which cluster in several analyses together and are of special interest. Detailed information on these subgroups can be retrieved from the NJ tree.

**Fig 2:**
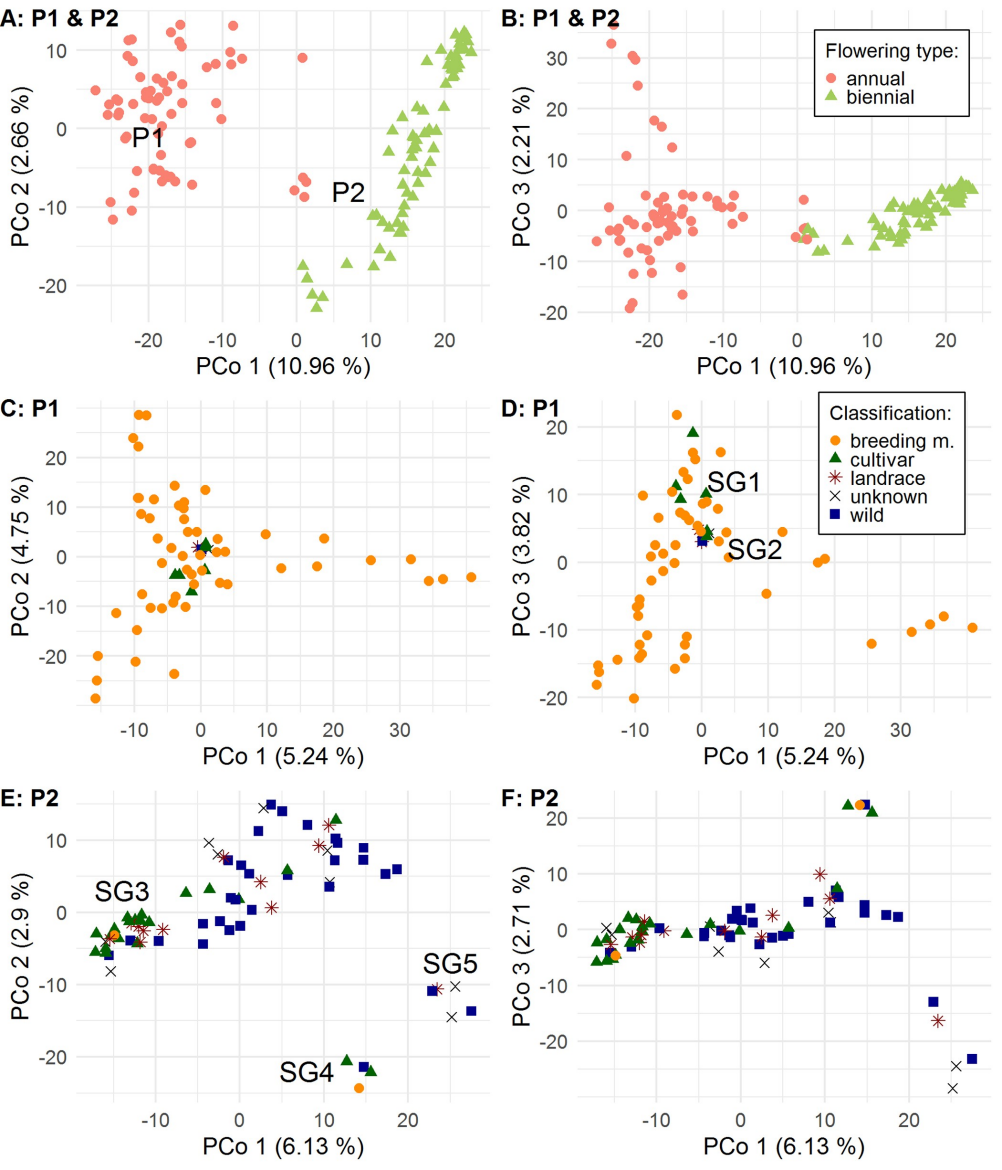
Principal coordinate analysis (PCoA) for 137 caraway accessions based on 13,155 SNP loci using Darwin software. (A) and (B) display PCoA for both subpopulations and discriminate flowering types. (C) and (D) display PCoA for subpopulation P1, containing all annual flowering types except SG4 and discriminate classifications. (E) and (F) display PCoA for subpopulation P2, containing all biennial flowering types and SG4 and discriminate classifications.

PCo1 and PCo2 explained 10.96% and 2.66% of the variance and PCo1 divided the accessions into two subpopulations defined as P1 and P2 (**Fig 2A and 2B**). P1 (n = 62) merely contains annual accessions and P2 (n = 75) contains all biennial accessions and a group of five annual accessions. Based on this result, we conducted separate PCoA for P1 and P2 to get deeper insights into the substructure of these subpopulations.–PCo2 shows that one of those five annual accessions is closely related to some annual accessions of P1 (**Fig 2A**). Therefore, this accession was analyzed with P1. –

P1 predominantly contains breeding material (n = 53). Only a few accessions are cultivars, landraces, wild material or are unclassified (n = 10). On PCo3 **(Fig 2D**), these accessions tend to split into two subgroups (SG1 and SG2), which is clearer on PCo4 (not shown). SG1 contains German cultivars (n = 4), including several breeding lines of the JKI, whereas SG2 includes all accessions of P1 originating from other countries (n = 8).

The PCoA for P2 shows an accumulation of most cultivars in the left end on PCo1 (**Fig 2E and 2F**), further on defined as SG3. The interesting subgroup SG4 of four annual accessions, including the Czech cultivars 'Aprim' and 'Ol-Alfa', is separated from the larger cluster of P2 accessions. This is also true for the subgroup SG5, which consists of biennial accessions originating from Russia, India and China.

**Fig 3:**
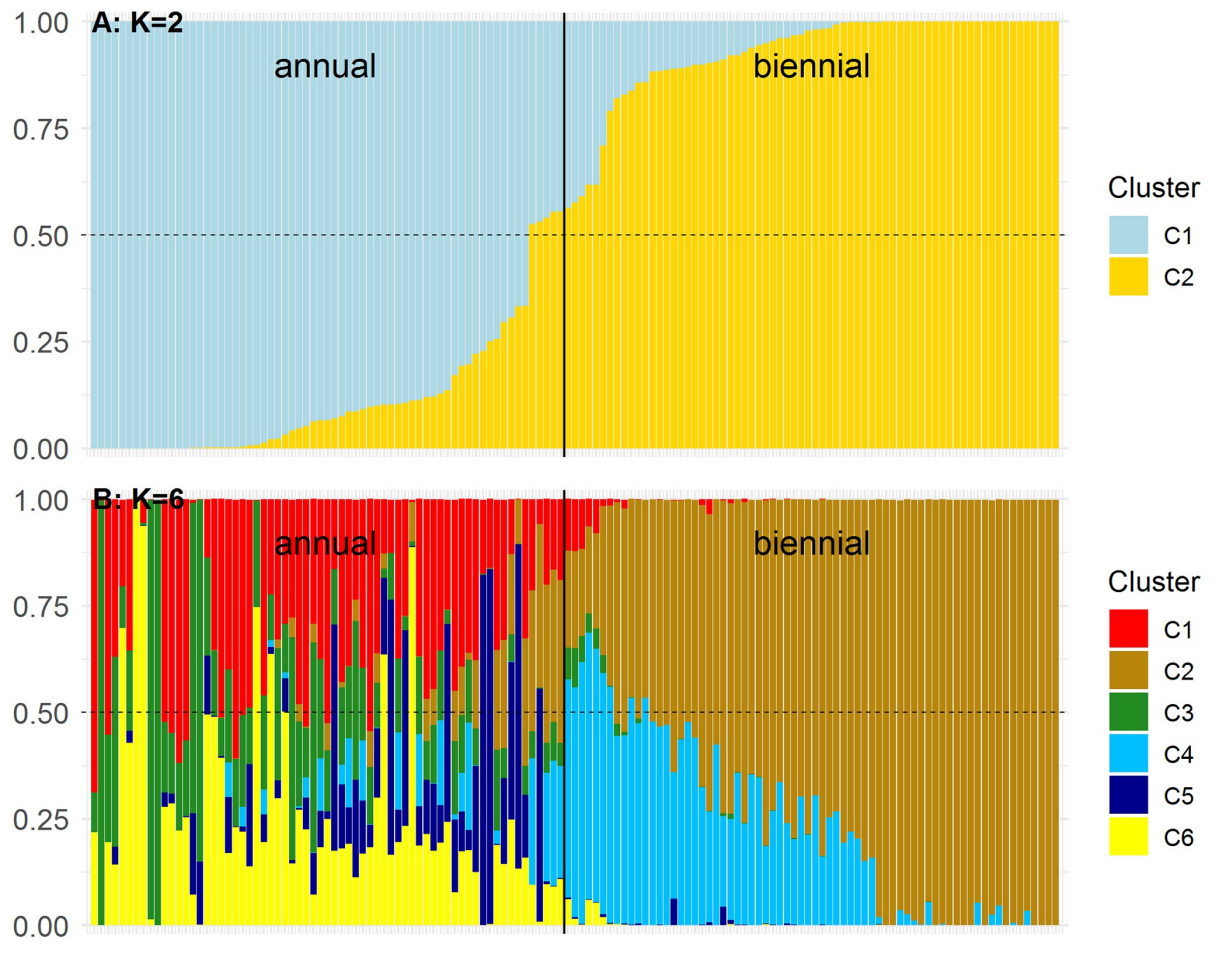
Estimated membership coefficients of 137 caraway accessions based on 13,155 SNP loci using STRUCTURE software. For (A) K = 2 and (B) K = 6. Accessions are sorted by decreasing membership coefficient for C1 (K = 2). The dashed line indicates the threshold of 0.5.

STRUCTURE results revealed two equally high ΔK-value peaks, for K = 2 and for K = 6 (**S1 Fig**). For K = 2, 62 accessions were assigned to subpopulation (or cluster) C1 and 75 accessions to C2 (membership coefficient > 0.5) (**Fig 3A**). This assignment is congruent with the assignment based on PCoA. For K = 6, twelve, sixty-one, six, nine, six and eight accessions were assigned to subpopulation C1-C6 (membership coefficient > 0.5), respectively (**Fig 3B)**. In total, 35 accessions could not be assigned to a subpopulation and were defined as an admixed group. Most biennial accessions (n = 61), including SG3, were assigned to C2 and all other biennial accessions (n = 9), including SG5, to C4. The assignment to C1 contains the annual accessions of SG1. Accessions assigned to C3, C5 or C6 form groups of related annual inbred lines. However, the majority of annual accessions could not be assigned to a subpopulation and were assigned to the admixed group. No assignment contains annual as well as biennial flowering types. Therefore, STRUCTURE results for K = 6 basically subdivide the subpopulations retrieved from K = 2. These subdivisions can be observed by PCoA as well (**Fig 2**).

To estimate the genetic variation explained by subpopulations, AMOVA was conducted for the subpopulations identified by STRUCTURE (K = 2 and K = 6). For K = 6, we analyzed the admixed group as a seventh subpopulation. In both cases (K = 2 and K = 6), genetic variation between subpopulations was low (7.84% and 7.65%, respectively).

**Fig 4:**
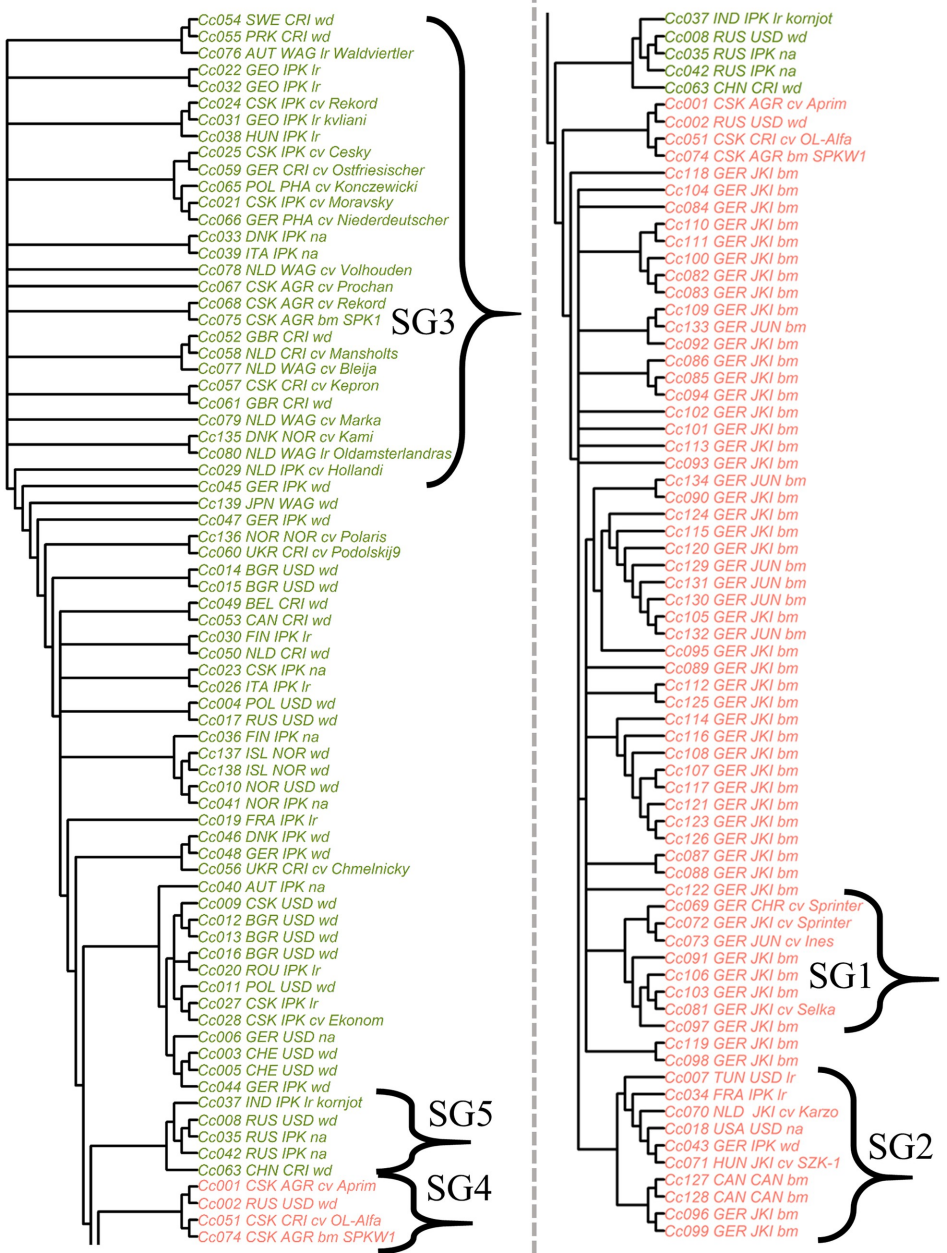
Neighbor joining tree for 137 caraway accessions based on 13,155 SNP loci, built using the BIONJ algorithm and a Euclidian distance matrix. Nodes which were supported below 30% by bootstrapping were dissolved to multifurcations. Green = biennial flowering types, red = annual flowering types. For better visibility, the tree was parted into two rows. On the left, there are all accessions of P2. On the right, there are all accessions of P1 with an overlapping part from P2.

For a better visualization of the genetic relations between single accessions, we built phylogenetic trees. A hierarchical clustering (UPGMA), a simple NJ and an improved NJ algorithm (BIONJ) were tested to build phylogenetic trees. The best correlation between the Euclidian distance matrix and a phylogenetic tree was found for the BIONJ tree (**Fig 4**; r = 0.94; for comparison: simple NJ, r = 0.86 and UPGMA, r = 0.79). The BIONJ tree also displays a clear partition between annual and biennial flowering types although the annual subpopulation emerges as a subgroup of the biennial subpopulation. SG4 appears as the nearest subgroup of annual flowering types to the biennial subgroup SG5. We also recover the biennial subgroup SG3, which contains most biennial cultivars. The BIONJ tree clearly separates German annual cultivars (SG1) from all annual accessions from other countries (SG2).

The neighbor network (**S2 Fig**) supports the partition into two subpopulations correlating with flowering type. SG4 and SG5 appear as rather intermediate subgroups between P1 and P2. However, net structures towards biennial accessions seem to be stronger. In general, net structures are prominent at the basis of subpopulations but are low between individuals within subpopulations. Branches of individual accessions are long, indicating that a lot of genetic diversity is stored on individual level.

**Fig 5:**
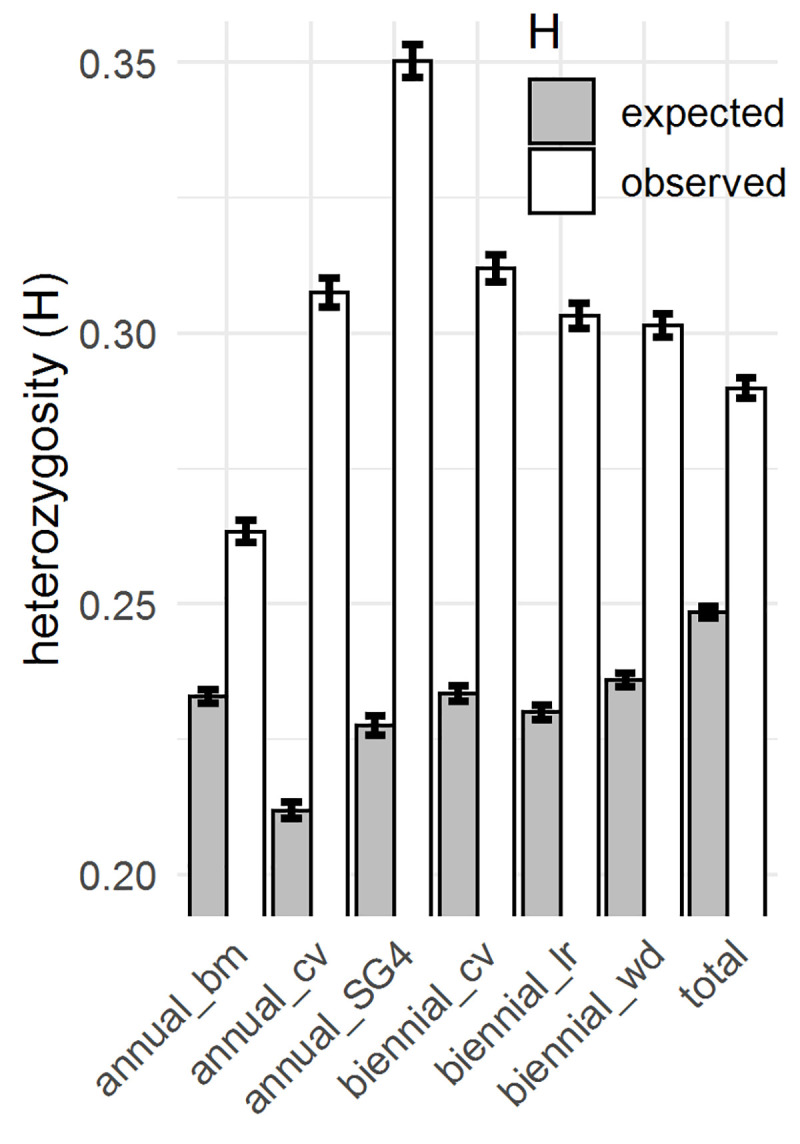
Observed (H_O_) and expected heterozygosity (H_E_) measured across 13,155 SNP loci for various subsets of caraway accessions. Subsets: 1. Annual breeding material, n = 53. 2. Annual cultivars, n = 6. 3. Annual subgroup SG4, n = 4. 4. Biennial cultivars, n = 19. 5. Biennial landraces, n = 12. 6. Biennial wild material, n = 29. 7. All 137 accessions including non-classified accessions. Error bars = standard errors.

H_O_ and H_E_ were used as estimators for genetic diversity. Primarily, we wanted to compare the genetic diversity of different classifications within the annual and biennial subpopulations.–The special annual subgroup SG4 was analyzed separately–. In addition, we wanted to survey whether biennial material was in fact highly heterozygous compared to annual inbred material. For all given subsets of accessions, H_O_ was higher than H_E_ (**Fig 5**). The H_O_ of the annual breeding material, consisting of 27 S4 lines, 24 S5 lines and two DH lines, (26.34%) was significantly lower than the H_O_ of annual cultivars (30.75%) (t = -24.404, df = 13154, p-value < 0.001). In contrast, the H_E_ was significantly higher (2.11%) in annual breeding material (t = 18.477, df = 13154, p-value < 0.001). The highest H_O_ was found in the annual subgroup SG4 with 35.03%. No larger differences in H_O_ or H_E_ were found between biennial cultivars, biennial landraces and biennial wild material. In general, the H_O_ of biennial material is on the same level as the H_O_ of annual cultivars. The lowest H_O_ (20.93%) among all accessions was found in a Canadian DH line (ID: 127) and the highest H_O_ (39.91%) in a wild Russian accession (ID: 002). Single values for heterozygosity per accession are listed in **S1 Table**.

## Discussion

We investigated a broad collection of caraway by GBS to reveal population structure and genetic diversity in caraway germplasm. We applied distance-based and model-based methods, which mutually supported our findings. Different types of visualization may enable the reader to retrieve general and detailed information on the relations between the 137 analyzed caraway accessions. We found a strong correlation between the upper most level of population structure and the flowering type. Therefore, we focused on this phenomenon for further analysis and in the following discussion. Nonetheless, we will also discuss some interesting findings within the substructures of the subpopulations. In addition, we were interested in the genetic diversity of cultivars compared to present breeding material and wild accessions.

### Flowering type

The flowering type was assessed by a field trial sown in spring. We defined accessions that flowered in the year of sowing as annual and in the year after sowing as biennial. Biennial flowering types need a cold stimulus (vernalization) for flowering induction [1,4]. Therefore, we distinguished precisely between accessions with (biennial) and without (annual) vernalization requirement. By this definition, we neglected the winter-annual ecotype. The Czech cultivars 'Aprim', 'Ol-Alfa' and the breeding material 'SPKW1' were bred for winter-annual growing and are therefore typically sown in autumn. However, these accessions lack a vernalization requirement. In addition, some genebank accessions with vernalization requirement are described as winter-annual ecotypes according to passport data (**S1 Table**).

In crossing experiments between annual and biennial material, annual flowering was found to be dominant [4]. Since most annual breeding material in our set originated from crossings between annual and biennial material, there could be a minor allele frequency of the recessive biennial allele. Weglarz [4] suggested that the flowering time could be controlled by a major locus and a few minor loci. We suggest that this major locus controls vernalization. Interestingly, the genetic control of vernalization was investigated for *Arabidopsis thaliana* and cereals [51–53]. These findings might be valuable to search for a vernalization locus in caraway.

### Genome size

Genome size was investigated by flow cytometry with *Solanum lycopersicum* cultivar 'Stupické' as well accepted reference standard [21,54,55], which resulted in an estimated genome size of 4.282 pg/2C (1C = 2.094 Gb using the conversion factor of 1 pg = 0.978 Gb [30]). In contrast, Das [19] published a 4C DNA content of 19.051pg (9.526 pg/2C) using Feulgen cytophotometry. This is more than twice the value found in this study. Doležel and Greilhuber [21] pointed out that flow cytometry is not precise enough to estimate the exact genome size; however, slight corrections of the reference standards will be available in the future. Nevertheless, the imprecision should be marginal compared to the difference between our findings and the findings of Das [19]. The large difference might lead to the assumption that Das [19] used an accession with a higher ploidy level, i.e., a tetraploid karyotype. In literature, autotetraploid material generated by colchicine treatment is described [56], but no polyploid caraway accession is known from nature. Anyway, the *C*. *carvi* var. KBI 1434 used by Das [19] is described as diploid karyotype, so that we cannot explain the discrepancy by differences in ploidy. Greilhuber [57] reports a twofold overestimation of genome size by several studies using Feulgen cytophotometry. This is congruent with our findings.

For a set of 35 caraway accessions, no considerable intraspecific variation was found. To the best of our knowledge, *Daucus carota*, *Coriandrum sativum* and *Apium graveolens* are the nearest relatives of *C*. *carvi* with an available reference genome (Estimated genome sizes: 1C = 0.473 Gb, 2.13 GB and 3.18 Gb, respectively) [58–60]. Furthermore, the genome size varies a lot in the Apiaceae family. Studies dealing with flow cytometry for species of the *Daucus* genus, *Eryngium duriaei* subspecies and diploid *Seseli libanotis* reported genome sizes from 0.92 pg/2C to 3.019 pg/2C [25]; 5.52 pg/2C to 6.2 pg/2C [26] and 2.592 pg/2C to 3.085 pg/2C [24], respectively. Genome duplication during speciation (paleopolyploidy) could be an explanation for the differences in genome size [61,62]. Indeed, Li *et al*. [59] assume a genome duplication event for celery after the split of Apiaceae from other taxa.

### SNP discovery and heterozygosity

As expected, GBS enabled the discovery of a large number of SNPs, suitable for a variety of downstream applications. For species for which a reference genome is available imputation of missing values can be a valuable method to improve the SNP data quality [18]. However, imputation was not possible for our data because the clustering merely generated contigs of random order.

As expected, annual breeding material, which mainly consists of advanced generation breeding lines, showed the lowest H_O_. However, Ho of 26.34% is much higher than theoretically expected for lines of the 4^th^ or 5^th^ inbreeding generation. Checking the frequency of heterozygous SNP calls per genotype in the raw SNP data reveals that it is much lower and that it is increased by the MAF filter applied. Nevertheless, the frequency of heterozygous SNPs per genotype is yet higher than expected for these advanced generation breeding lines. Selection mechanisms and/or unintended outcrossing could be reasons for this. Partially, the existence of multiple copies of highly similar but non-allelic sequences might lead to heterozygous SNPs [18,63,64]. We assume that such heterozygous loci would occur randomly per genotype and that all genotypes would be affected. Therefore, we anticipate that such loci would not severely affect the results for population structure. In general, this issue has to be addressed in more detail, in particular, if SNP data will be used for downstream applications like GWAS or the development of diagnostic markers.

### Population structure and genetic diversity

#### Subpopulations P1 & P2

Bayesian clustering analysis and PCoA revealed an optimal number of two subpopulations, which differ in flowering type of accessions (**Figs 2A, 2B and 3A**). This indicates that the flowering type causes the main structure in the population of 137 caraway accessions under investigation. In addition, STRUCTURE results also support the presence of six subpopulations, which show differentiation within the annual (P1) and biennial (P2) subpopulation (**Fig 3B**). In general, occurrence of two peaks of K = 2 and K = 6 is not contradictory. K = 2 describes the upper most level of population structure caused by the flowering type, whereas K = 6 describes a lower level of population structure, which is also indicated by PCoA.

The correlation between population structure and flowering type is well supported. Yet, genetic differentiation between subpopulations is rather low (AMOVA). We hypothesize that both subpopulations mainly differ in the genomic region of the locus controlling vernalization, whereas in most other genomic regions, both subpopulations could be similarly variable. This might be attributable to the fact that most annual breeding lines originate from crossings between annual and biennial breeding material.

#### Annual subpopulation P1

Those crossings, mentioned above, aimed to bring a higher essential oil content of biennial breeding material into the annual breeding pool. During multiple steps of inbreeding, annual flowering and high essential oil lines were selected. Hence, we expected a large intermediate group, but instead most breeding lines cluster nearby original annual accessions (the cultivar 'Sprinter' comes closest to the parental annual material). Therefore, the selection towards annual flowering might have had the largest impact on the genetic constitution of the breeding material. As discussed above, this selection might only affect a small genomic region because little variation is explained by the separation into subpopulations in accordance with flowering type. *Vice versa*, alleles of the biennial parents, which promote high essential oil content, were not strongly selected. We assume that alleles for higher essential oil content were already present in parental annual material used for crossings. Indeed, breeding history at the JKI shows that recurrent selection within original annual breeding material could increase the essential oil content in the same way as crossings between annual and biennial breeding material [65].

According to Pank [1], annual caraway is indigenous to the Middle East and the East Mediterranean region. Furthermore, it is assumed that there are wild or escaped annual caraway populations in North African countries like Tunisia [7,66]. Németh [3] assumed an Egyptian origin of European annual cultivars. Laribi *et al*. [7] found that a German accession was closer linked to an Egyptian accession than to Tunisian accessions. First annual cultivars for the cultivation in Europe were released in Hungary ('SZK-1') and the Netherlands ('Karzo') [1]. Toxopeus [56] reports that 'Karzo' was produced by crossings between annual accessions from Hungary, Poland and Egypt. The PCoA and the BIONJ tree indicate a close relationship between those cultivars and gene bank accessions which are located in SG2 (**Figs 2C, 2D and 4**). This subgroup contains 'SZK-1' and 'Karzo' as well as Tunisian, German, French, Canadian and North American accessions.–The German accession, which is classified as “wild”, has to be escaped from cultivation or is wrongly classified.–The close relationship indicates an identical origin and an active exchange of annual material between breeders, farmers and gene banks. German cultivars seem to belong to a separate but related subgroup (SG1). Thus, there might have been a slightly different origin or strong selection.

The H_E_ was significantly higher for annual breeding material than for annual cultivars (**Fig 5**). However, due to different numbers (n = 53 to n = 6), both groups are scarcely comparable. To the best of our knowledge, only four annual cultivars ('Springcar', 'Balady', 'CN-1' [1] and 'Moran') are missing in our set. Therefore, in general, the number of annual cultivars is low. In future breeding, the genetically diverse breeding material can extent the gene pool of annual cultivars.

#### Annual subgroup SG4 of subpopulation P2

Pank [1] reports the Czech variety 'Alpha' to be a transient variety between annual and biennial flowering types with reduced vernalization requirement. Most likely 'Alpha' is closely related with the recently bred Czech cultivar 'Aprim' (Smirous, P. 2020, Agritec Ltd., personal communication) and the accession 'Ol-Alfa'. 'Ol-Alfa' and 'Aprim' are rather located with two other annual accessions in the otherwise biennial subpopulation P2 (**Figs 2A, 2B and 3A**). Literature provides no more information to reconstruct the breeding history, but breeders state that some annual breeding material was crossed and backcrossed for several times with biennial material (Nemeth, E. 2020, personal communication). 'Aprim' most likely originates from these breeding schemes. Several backcrossings would perfectly explain the assignment to the biennial subpopulation. The appearance of an annual Russian accession classified as “wild” in this subgroup is enigmatic (**Fig 4**). It might be the most parsimonious way to assume a wrong classification. Alternatively, we could assume a distinct (Russian) origin of 'Alpha' and 'Aprim', but there are no breeding reports that support this thesis. Although ‘Aprim’ was bred for winter-annual growing season with sowing in August, as mentioned above, it was sown in spring and no vernalization requirement was observed. This subgroup is a valuable gene pool for breeding of winter-annual caraway showing an outstanding frost-resistance. In summary, we can report that winter-annual ecotypes (without vernalization requirement) are more closely related to biennial ecotypes than to summer-annual ecotypes.

#### Biennial subpopulation P2

The biennial subpopulation P2 contains many more accessions classified as wild than P1 and contains geographically diverse caraway accessions. The subgroup SG5 contains three Russian, one Indian and one Chinese accession (**Fig 4**). Having a closer look at the STRUCTURE results for K = 2 or K = 6, SG5 contains "annual clusters" to a larger proportion (**Fig 3A and 3B**) and appears in all investigations as the closest subgroup to annual accessions (**Figs 2A, 2B and 4**). In nature, these accessions might overlap geographically with or be related to an annual population in the Middle East, whose extensions to the north and west are unknown. Accessions from Japan and North Korea do not belong to that subgroup, so that we should not define SG5 as an Asian subgroup (**Fig 4**). In addition, we found some wild (or unclassified) accessions of nearby geographic origin that cluster in one subgroup, for instance, five Scandinavian accessions (**Fig 4**). However, some wild accessions seem to be randomly distributed regarding their geographic origin (**Fig 4**). We can only speculate about reasons. In some cases, passport data might be incorrect. Alternatively, wild accessions might originate from escaped plants, which were not indigenous in a country.

The accumulation of biennial cultivars in SG3 (**Figs 2E, 2F and 4**) indicates that a certain gene pool was preferred in caraway breeding. This subgroup contains cultivars from the Netherlands, Czech Republic, Germany, Austria, Poland and Denmark, indicating an active exchange between national breeding programs. It can be suspected that this subgroup keeps many advantageous alleles for agronomic traits, but untapped valuable alleles might exist in other subgroups as well. Wild accessions located in SG3 originate from Sweden, Denmark and Great Britain. This might lead to the assumption that the majority of improved biennial cultivars originated from North-West European wild material. However, North European wild material (from Denmark, Norway, Island and Finland) appears outside SG3 as well. A more expanded sampling in wild caraway populations would be necessary to get reliable insights into the domestication history of biennial caraway. However, there probably is more than one domestication event.

The differences in H_E_ between biennial cultivars, landraces and wild accessions are marginal. Thus, we cannot confirm a decrease of genetic diversity due to domestication and breeding. However, only a subset of wild accessions was included in the set of accessions under investigation. Therefore, it is difficult to evaluate the genetic diversity of wild caraway.

## Conclusions

As expected, GBS turned out to be a robust method to reveal genetic diversity and population structure in caraway. In particular, for minor crops, such as most spices and medicinal plants, GBS or similar methods of NGS could be the method of choice to move these crops into the age of molecular genetic research and breeding.

The analysis of population structure revealed the partition into an annual and a biennial subpopulation. A small group of winter-annual accessions is more closely related to biennial accessions. The genetic differentiation between subpopulations, however, is low. Therefore, many alleles which are favorable for breeding might be present in the annual and biennial gene pool. Phenotypic evaluation has to show whether genetic transfer between both gene pools is necessary yet for future breeding programs under changing climatic conditions.

Future breeding programs should benefit from the use of marker-assisted selection (MAS). For this purpose, SNP loci from GBS can be easily converted into diagnostic markers. Further investigations are necessary to identify SNP loci associated with flowering type and other traits of interest.

## Supporting information

S1 Fig Delta K values from STRUCTURE analysis of 137 caraway accessions based on 13,155 SNP loci. For one to ten assumed subpopulations based on STRUCTURE results processed by STRUCTURE harvester.
(TIF)

S2 Fig Neighbor network for 137 caraway accessions based on 13,155 SNP loci using SplitsTree software. (TIF)

S1 Table Overview of 137 caraway accessions used in GBS analysis. This table provides detailed information on passport data and gathered data of all 137 accessions.
(XLSX)
