# Supplementary figures and images for: On genetic diversity in caraway: Genotyping of a large germplasm collection
Source: PLoS One. 2020 Dec 29;15(12):e0244666. doi: 10.1371/journal.pone.0244666 (PMC7771672; doi:10.1371/journal.pone.0244666)

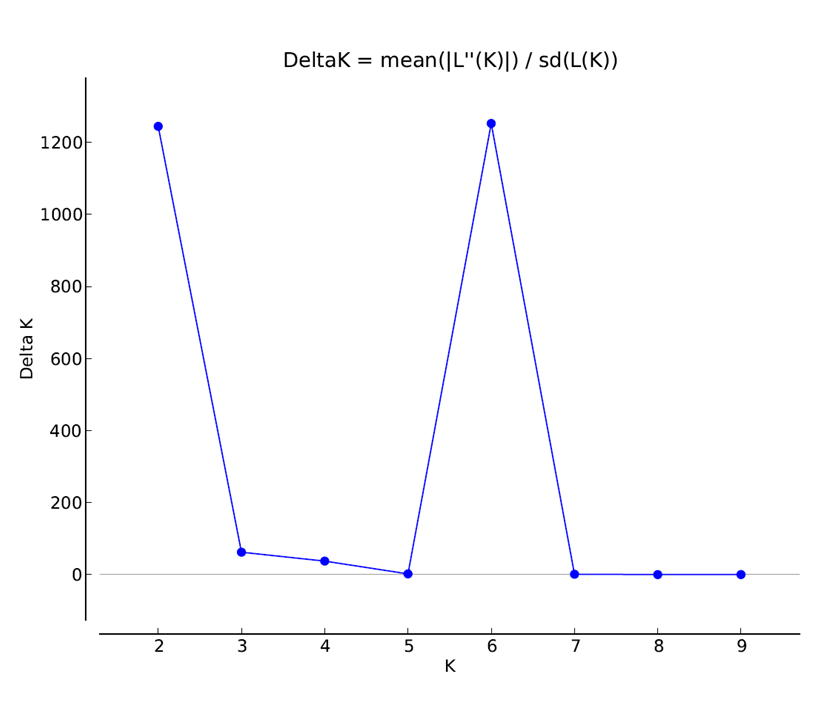

Supplement: S1 Fig — For one to ten assumed subpopulations based on STRUCTURE results processed by STRUCTURE harvester. (TIF) [file pone.0244666.s001.tif]

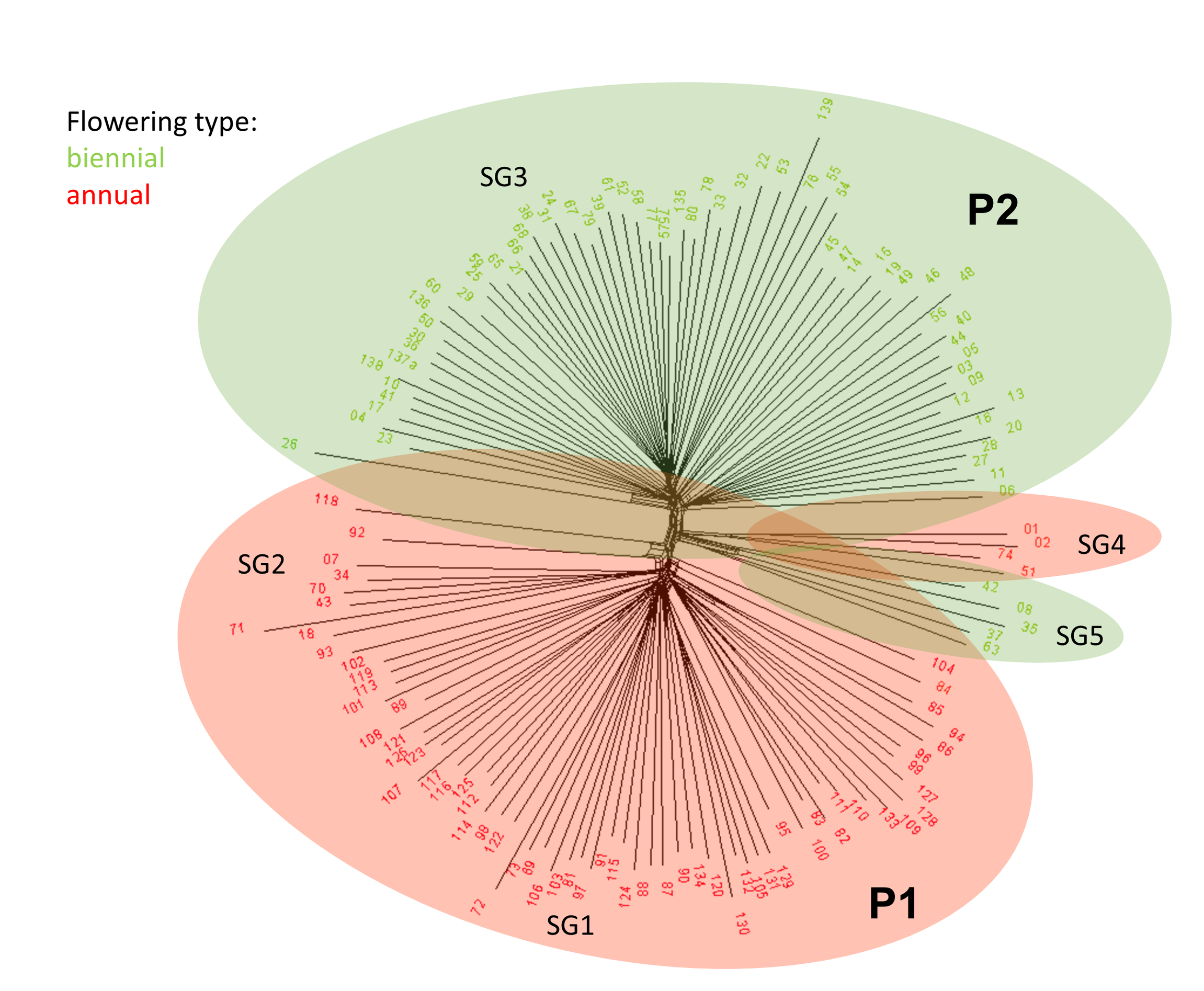

Supplement: S2 Fig — (TIF) [file pone.0244666.s002.tif]
